# Expression of Concern: RNAi-Dependent and Independent Control of LINE1 Accumulation and Mobility in Mouse Embryonic Stem Cells

**DOI:** 10.1371/journal.pgen.1005377

**Published:** 2015-06-29

**Authors:** The PLOS Genetics Editors

Concerns have been raised regarding some of the data in the *PLOS Genetics* article “RNAi-Dependent and Independent Control of LINE1 Accumulation and Mobility in Mouse Embryonic Stem Cells”, notably in panel A in Fig 4, in panels A and F in S4 Fig, and with the statistical analyses used to produce Fig 2. The authors have responded to these concerns, acknowledging that some errors were made in figure preparation and with some statistical tests, however a final resolution has not yet been reached, and the matter is being evaluated by the authors’ institution.

This Expression of Concern should not be considered as a statement regarding validity of the work but rather as a notification to readers, and an intent to provide additional information as it becomes available.
